# Dual Contraceptive Method Utilization and Associated Factors Among HIV Positive Women Attending ART Clinic in Finote-Selam Hospital: Cross-Sectional Study

**DOI:** 10.1007/s10508-023-02593-8

**Published:** 2023-04-07

**Authors:** Anteneh Jemberie, Bewket Yeserah Aynalem, Liknaw Bewket Zeleke, Addisu Alehegn Alemu, Tenaw Yimer Tiruye

**Affiliations:** 1 Health Science College, Debre Markos University, Debre Markos, 269 Ethiopia; 2 School of Women’s and Children’s Health, University of New South Wales Sydney, Kensington, Australia; 3 Cancer Epidemiology and Population Health Research Group, Allied Health and Human Performance, University of South Australia, Adelaide, Australia

**Keywords:** Dual contraceptive utilization, Women living with HIV, Finote Selam Hospital, Ethiopia

## Abstract

Worldwide, the human immune deficiency virus is the leading cause of death for women of childbearing age. Around two-thirds of all pregnant women living with the human immune deficiency virus experience an unintended pregnancy. The correct and consistent use of dual contraceptive methods is important to prevent unintended pregnancy and transmission of sexually transmitted infections. However, little is known about the utilization of dual contraceptive methods among HIV-infected women. Thus, this study aimed to assess dual contraceptive utilization and associated factors among HIV-positive women attending antiretroviral therapy (ART) in Finote Selam Hospital, Northwest Ethiopia. Facility-based cross-sectional study design was conducted from September 1 to October 30, 2019, in Finote Selam Hospital among HIV-positive women. A systematic random sampling technique was used to select study participants and the data were gathered by an interviewer-administered structured pretested questionnaire. Factors associated with dual contraceptive use were identified through binary logistic regression. Finally, a *p*-value < 0.05 was taken as a cutoff point to declare a significant association, and the direction and strength of the association were determined by the adjusted odds ratio. The study showed that 21.8% of HIV-positive women attending ART care in Finote Selam Hospital utilize dual contraceptive methods. Dual contraceptive utilization was significantly associated with having a child (AOR: 3.29; CI 1.45, 7.47), having family support to use dual contraceptives (AOR: 3.02; CI 1.39, 6.54), having multiple sexual partners (AOR: 0.11; CI 0.05, 0.22), and urban residence (AOR: 3.64; 1.82, 7.3). The study revealed that low utilization of dual contraceptive methods. This will continue major public health problems in the study area unless future interventions conducted.

**Supplementary Information:**

The online version contains supplementary material available at 10.1007/s10508-023-02593-8.

## Introduction

Globally in 2017, about 33 million people were living with HIV/AIDS and HIV is the leading cause of death for women of childbearing age (Singh et al., 2010; World Health Organization, 2011). Unprotected sex among people living with HIV is a challenging issue in HIV prevention. More than two-thirds of them were living in sub-Saharan Africa and close to three-fourths of all AIDS-related deaths occurred in this region (Berer, 2006; Wilson et al., 2003). Nearly 92% of pregnant women living with HIV were also from sub-Saharan Africa (Ngure et al., 2009). According to the 2016 Ethiopian Demographic Health Survey, the prevalence of HIV infection in women was 1.2% (Central Statistical Agency & ICF, 2017).

Unintended pregnancy mainly results from engaging in vaginal sexual activity without the use of contraception, or due to incorrect use of a contraceptive method, but may also arise from the failure of the contraception method when used correctly to prevent pregnancy (Oulman et al., 2011). Unintended pregnancy, births, and STIs could have negative consequences for women, children, families, and society at large (Darega et al., 2014). The rate of unwanted pregnancy was found to be significantly higher in HIV-positive women than in their counterparts 20.7% versus 13.5% (Warren, 2013).

HIV/AIDS was of major global health importance; in Sub-Sahara Africa, 60% of people living with HIV/AIDS and more than half of these population groups were females (World Health Organization, 2011). HIV-infected women of childbearing age are also highly vulnerable to sexually transmitted infections (STIs) (Aboud et al., 2008). High-risk sexual behavior in patients on highly active antiretroviral therapy (HAART) is a major social and public health problem. If HIV-positive individuals continue to have unprotected sex with HIV-negative persons or persons of unknown HIV status, inconsistent condom use behavior may continue to spread HIV infection (Campbell et al., 2009; McClelland et al., 2006).

The co-occurrence of HIV and unintended pregnancy has prompted a relatively recent body of work on dual protection, simultaneous protection against STIs, and unintended pregnancy (Berer, 2006). Some studies that have considered the benefits of dual protection for people living with HIV show that dual protection can be an effective strategy to prevent HIV transmission to partners and to promote safe childbearing; but little of it is known in our country (Heffron et al., 2010; Ngure et al., 2009).

The World Health Organization recommends that women living with HIV use dual contraceptive methods or dual protection to prevent unintended pregnancies and STIs (Munsakul et al., 2016). Dual contraceptive utilization refers to the use of a barrier contraceptive plus another modern family planning method that can prevent pregnancy (Wilson et al., 2003). Dual contraceptive methods have paramount benefits for HIV-positive women to control mother-to-child transmission and partner infection. (Heffron et al., 2010; Ngure et al., 2009). The promotion of dual protection plays an important role in public health interventions (Myer et al., 2002).

In Ethiopia, over 90% of adult cases of HIV are attributable to heterosexual activity*.* For many people living with HIV in the country, ART enables them to return to normal life, including a resumption of sexual activity and a desire for children. Unless appropriate care is taken in sexual activity and the desire to have children, it also means for HIV-infected women that the chances of transmitting the infection to their children and their partner are higher (Central Statistical Agency [Ethiopia] & ICF International, 2012).

## Method

### Participants

A facility-based cross-sectional study design was conducted. The study was conducted from September 1 to October 30, 2019, among sexually active reproductive-age women on ART in Finote Selam Hospital. The hospital is found in Finote Selam town which is located 385 km northwest of Addis Ababa and 185 km to the south of Bahirdar, the capital of Amhara National Regional State. The hospital serves more than 700,000 people in the catchment area. During data collection, there were 1912 HIV-positive clients on ART of which 623 were sexually active reproductive-age women.

All HIV-positive women who were attending ART in Finote Selam Hospital were the source population and HIV-positive sexually active reproductive-aged women attending ART during the study period were the study population. HIV-positive women, who were unable to communicate verbally or were seriously ill at the time of data collection, were excluded from the study.

The sample size was calculated by considering single population proportion and double population proportion formulas, and finally, the highest sample size, 303, yielded by the single population proportion formula was used. In the single population proportion formula, the prevalence rate of dual family planning utilization was taken at 13.2% (Reta et al., 2019), and 95% confidence interval with 4% marginal error and 10% contingency for nonresponse rate.

A systematic random sampling technique was applied to select study participants from the total number of 623 HIV-positive reproductive-age women who were expected to visit the hospital for ART service during the data collection period. Sexually active women were identified through prescreening. The sampling interval was determined by the formula K = N/n, where K refers to the sampling interval, N refers to the total number of sexually active HIV-positive women who are expected to visit the hospital for ART care during the data collection period and n refers to the sample size. The first sample to be included in the study was obtained using the lottery method, and then we can pick every k value until the required sample size was obtained.

### Measures

#### Dependent Variable

Dual contraceptive utilization (yes/no).

#### Independent Variable

- *Demographic variables* Age of the women, education, residence, ethnicity, religion, average monthly income, occupation.
- *Sexual and reproductive related variables* Age at first sex, have had child, number of children, desire to have more child, partner desire to have more child, sex with whom use a condom, the reason for using a condom, the experience of spousal abuse, women’s decision-making autonomy, support to use dual contraceptive.
- *HIV-related variables* Duration of ART, partner HIV test, CD4 count.

#### Operational Definition

- *Dual contraceptive utilization* refers to the HIV-positive reproductive age, women who used two methods of contraception simultaneously, a barrier method (condom) and other modern contraceptive methods (pills, injectable, Norplant/implant, and IUCDs) (Wilson et al., 2003).
- *Sexually active women* refers to reproductive-age women who could have sexual intercourse during the last 4 weeks before the survey (Central Statistical Agency & ICF, 2017).

### Procedure

Two BSc-holder midwives collected the data through face-to-face interviews using interviewer-administered pretested structured questionnaires under the supervision of an MSc-holder midwife. The respondents were asked before while they were waiting to receive the service, taking them to a private room.

To maintain the quality of data, supervisors and data collectors received a one-day training language, and the consistency of the tool was checked and pretested before commencing data collection. The supervisors closely supervised the data collectors and the principal investigator was available for further guidance while the data collection. The supervisor and the investigators checked the completed questionnaire daily for completeness and inconsistency.

### Data Analysis

The collected data were entered into EPI data version 3.1 statistical software and then exported to SPSS version 20 for data cleaning and analysis. Descriptive statistics were computed to describe the study population through relevant variables. Binary logistic regression was used to identify factors associated with dual contraceptive method utilization. Independent variables that showed a *p*-value ≤ 0.25 in the bivariate logistic regression analysis were included in the multivariable logistic regression analysis. Finally, variables with *p*-value < 0.05 at a 95% confidence interval were declared as significantly associated with dual contraceptive method utilization. The strength & direction of the association was interpreted based on the adjusted odds ratio.

## Results

### Demographic Characteristics

All intended 303 sexually active women who were on ART participated in the interview giving a response rate of 100%. The mean age of the study participants was found to be 35.2 (SD ± 5.2) and the largest proportion (53.1%) of the study respondents were in the age group of 30–39. The majority (60.7%) of the study respondents were rural dwellers and regarding educational status, nearly one-third (32.7%) of the participants attended a high school program. The mean/average income of the study participants was 2027 Birr (Table 1).

**Table 1:** Demographic characteristics of HIV positive women attending ART Clinic in Finote-Selam Hospital, Northwest Ethiopia, 2019

Variables	Category	Frequency	Percent
Age groups	18–29	46	15.2
	30–39	161	53.1
	40–49	96	31.7
Marital status	Married	108	35.6
	Single	89	29.4
	Divorce	61	20.1
	Widowed	45	14.9
Ethnicity of mothers	Amhara	248	81.8
	Oromo	30	9.9
	Tigray	7	2.3
	Other	18	5.9
Religion	Orthodox	257	84.8
	Muslim	37	12.2
	Protestant	9	3
Residence	Urban	119	39.3
	Rural	184	60.7
Educational status	Cannot read and write	29	9.6
	Read and write	38	12.5
	Elementary	75	24.8
	Highschool and preparatory	99	32.7
	College and above	62	20.5
Occupation	Housewife	78	25.7
	Merchant	117	38.6
	Government employ	74	24.4
	Daily laborer	34	11.2
Average monthly income	< 1000 Birr	30	9.9
	1001–2000 Birr	164	51.4
	2001–3000 Birr	66	21.8
	> 3001 Birr	43	14.2

### Sexual and Reproductive-Related Factors

More than half (57.8%) of the participants experienced their first sex before 18 years old. All respondents had sexual intercourse in the last month of which the majority (64.7%) had multi-sexual partners. Regarding the decision-making autonomy of the respondents, 60.4% were involved in all decisions regarding their health care, major household purchases, and visits to their family or relatives. Around one out of three (34.3%) participants experienced either physical or sexual spousal abuse (Table 2).

**Table 2:** Reproductive and sexual related factor characteristics of HIV positive women attending ART Clinic in Finote-Selam Hospital, Northwest Ethiopia, 2019

Variables	Categories	Frequency	Percent
Age at first sex	< 18 years	175	57.8
	> 18 years	128	42.2
Have had child	Yes	181	59.7
	No	122	40.3
Number children	One child	51	28.1
	2–4 child	115	63.6
	> 4 child	15	8.3
Desire to have more child	Yes	143	57.8
	No	160	42.2
Partner desire to have more child	Yes	89	29.4
	No	214	70.6
Sex with whom	Husband	108	35.6
	Multiple partners	195	64.4
Use condom	Yes	191	63.0
	No	112	37.0
Reason for using a condom	Prevent pregnancy	50	26.2
	To reduce viral load	15	7.9
	Fear of other STIs	36	18.8
	Health worker advice	90	47.1
Experience of spousal abuse	Yes	104	34.3
	No	199	65.7
Women’s decision-making autonomy	Yes	183	60.4
	No	120	39.6
Support for to use of dual contraceptive	Yes	183	60.4
	No	120	39.6

### HIV-Related Factor Characteristics

A total of 144 study participants' partners were tested for HIV test and none of them were negative. Nearly half (51.2%) of respondents started ART greater than 24 months before the study and the majority (60.1%) of the respondent's health status got improved after ART started (Table 3).

**Table 3:** HIV related factor characteristics of HIV positive women attending ART Clinic in Finote-Selam Hospital, Northwest Ethiopia, 2019

Variables	Category	Frequency	Percent
Partner HIV testing	Yes	144	47.5
	No	159	52.5
Length of ART initiation	< 12 month	34	11.2
	12–24 month	114	37.6
	> 24 month	155	51.2
Health status after ART initiation	Improved	182	60.1
	Same	97	32
	Worsened	24	7.9
CD4 count	250–350	18	5.9
	350–500	125	41.3
	> 500	46	15.2
	Do not remember	114	37.6

### Dual Contraceptive Utilization

The study showed that 66(21.8%) (95% CI 17.4–26.1%) the participants used dual contraceptive methods of which 45 (68.2%) study participants used Depo-Provera plus condom; 13 (19.7%) study participants used oral contraceptive pills plus condom and 8(12.1%) study participants used IUD plus condom.

About 181 respondents were having a child and of these 54 (29.8%) utilized dual contraceptive methods. Again, 144 respondents had a partner tested for HIV, and 39 (27%) of these utilized dual contraceptive methods (Table 4).

**Table 4:** Multivariate analysis of factors associated with dual contraceptive utilization among HIV positive women attending ART Clinic in Finote-Selam Hospital, Northwest Ethiopia, 2019

Category	Dual contraceptive utilization		COR (95% CI)	AOR (95% CI)
	Yes	No		
*Having child*				
Yes	54	127	**3.89 (1.98–7.66)**	**3.29 (1.45–7.47)**
No	12	110	1	1
*Having family support*				
Yes	49	134	**2.22 (1.21–4.1)**	**3.02 (1.39–6.54)**
No	17	103	1	1
*Partner HIV test*				
Yes	39	105	1.82 (1.04–3.16)	2.81 (0.80,9.21)
No	27	132	1	
*Sexual partner*				
Husband	45	63	1	1
Multiple partners	21	174	**0.17 (0.09–0.3)**	**0.11 (0.05–0.22)**
*Age at first sex in years*				
< 18	48	127	2.31 (1.27–4.2)	0.54 (0.09,3.65)
> 18	18	110	1	
*Residence*				
Urban	44	75	**4.32 (2.42–7.72)**	**3.64 (1.82–7.30)**
Rural	22	162	1	1
*Desire to have a child*				
Yes	13	130	1	
No	53	107	4.95 (2.56–9.57)	2.01 (0.93,4.46)

Bold values indicate the significant association in the multivariable logistic regression analysis

### Factors Associated with Dual Contraceptive Utilization

Factors associated with dual contraceptive method utilization were identified through binary logistic regression. Initially, bivariate logistic regression was conducted to examine the association of each independent variable with the outcome variable at a *p*-value of 0.25. Having a child, having family support, urban residence, partner, HIV test, having age at first sex, having multiple sexual partners, and respondents' desire to have a child were identified as potential factors of dual contraceptive utilization. In the multivariable logistic regression analysis, having a child, having family support to use dual contraceptive methods, urban residence, and multiple sexual partners were found to be significantly associated with dual contraceptive utilization with a *p*-value < 0.05. The participants who have a child were 3.29 (AOR: 3.29; CI 1.45, 7.47) times more likely to use dual contraceptives compared to having had no children. Participants who have family support use dual contraceptive methods 3.02(AOR: 3.02; CI 1.39, 6.54) times more likely than those who have no family support to use dual contraceptives. Regarding respondents, who have multiple sexual partners 89% (AOR: 0.11; CI 0.05, 0.22) less likely to use dual contraceptive methods compared to those who have a regular sexual partner. This study also showed that participants who were urban residents were 3.64 (AOR: 3.64; CI 1.82–7.3) times more likely to use dual contraceptives compared to rural residents (Table 4**).**

## Discussion

In this study, we investigated dual contraceptive utilization and associated factors among HIV-positive reproductive-age women. The study revealed that about one in every five HIV-positive women, 21.8% with (95% CI 17.4–26.1%) use dual contraceptives. The finding showed that there was low coverage of dual contraceptive methods utilization among women attending HIV clinics. However this result may help the concerned bodies to conduct the future interventions like health education and behavioral change. This in turn may also help those women who attend ART clinics to change their ability and attitude of using dual contraceptive methods throughout their reproductive life. Even though the finding of this study was low, it was higher when compared to the cross-sectional study conducted in Gondar Hospital, Amhara region, Ethiopia, on HIV-positive women which were 13.2% (Reta et al., 2019) and Kenya (10%) (Demissie et al., 2015). The difference might be due to poor social support, educational level, study period, awareness creation about dual contraceptive method utilization, and living conditions. However, the finding was similar within the cross-sectional study conducted in Gebretsadik Shawo Hospital, Keffa Zone, SNNPR, Ethiopia, on HIV-positive women which were (19.8%) (Meseret et al., 2015). However, this finding was lower than a study conducted in southern Ethiopia Hossana Hospital dual contraceptive utilization among HIV-positive reproductive age women attending ART clinic findings showed that (28.3%) use dual contraceptive utilization (Jifar et al., 2017). The factors like having a child, family support to use dual contraceptives, multiple sexual partners, and urban residence were significantly associated with dual contraceptive utilization. The participants who have a child were 3.29 times more likely to utilize dual contraceptives compared to have no children, this finding was similar to the study conducted in Fiche town, people living with HIV who have living children were ten times more likely to have had used dual contraceptive methods compared to have no living Children's (Demissie et al., 2015). The possible justification for this might be those respondents who have no child may need to have a child so that they may not be eager to use dual contraceptive methods.

According to this study, HIV-positive women were who have family support to use dual contraceptives 3.02 times more likely to use dual contraceptives than those who have no family support, This finding was similar to the cross-sectional study done in Gebretsadik Shawo Hospital, Ethiopia (Meseret et al., 2015). Other studies conducted in Uganda among HIV-positive women results showed that participants who reported approval of their spouse or family support were seven times more likely to use contraceptive use than those who reported no approval of their spouse/family support (Joseph, 2010). This might be because these women are expected to have more freedom to negotiate safer sex and birth spacing and the positive effects of open discussion on a couple’s dual contraception use have been widely demonstrated in different studies.

According to respondents who have multiple sexual partners, 89% reduced to use of dual contraceptives compared to having regular sexual partners (husband). This finding was similar to the cross-sectional study conducted in Fiche Hospital, where the HIV-positive women who had regular sexual partner intercourse were 4.9 more likely to use dual contraceptives utilization than compared to those with multiple sexual partners (Demissie et al., 2015). Another study conducted in India married HIV-positive reproductive-age women who used dual contraception to prevent the risk of transmission of HIV to their partner were three times more likely than their counterparts (Chakrapani et al., 2011). The possible explanation, for this reason, might be, the respondents who have a regular sexual partner may have more freedom to use dual contraceptive methods than respondents with multiple sexual partners. Another possible reason might be those who have multiple sexual partners may be exposed to different addictions which may make them not use dual contraceptive methods as compared with those who have regular sexual partners. Regarding respondents’ residence having urban residences were 3.64 times more likely to utilize dual contraceptives compared to rural residences. This finding was similar to the cross-sectional study done in Gebretsadik Shawo Hospital, HIV-positive women who came from rural areas were fewer dual contraceptive method users than those from urban areas (Meseret et al., 2015). Another study conducted in South Africa has also shown that living in an urban area was more strongly associated with the use of dual contraceptive methods than those who live in rural areas (Harvey et al., 2004). This variation may be attributable to the information gap between rural and urban areas and the accessibility of family planning services and logistics in urban than rural areas.

According to a study conducted in Gondar Hospital, counseled health care providers about dual contraceptive partners involved in posttest counseling and discussed with a partner dual contraceptives were significantly associated (Reta et al., 2019). However, these variables were not significant in this study. Possible reasons for this variation might be due to educational level, social support, discrimination and stigmatization, study area and period, awareness creation about dual contraceptive method utilization & health care provider counseling, and negotiation skills.

### Conclusion

The overall magnitude of dual contraceptive utilization is still low in this study, and service integration within the ART was low. This will be a great concern for the transmission of the virus from mother to babies and partners and the risk of complications following unintended pregnancy. This will continue with major public health problems in the study area unless future intervention focuses on barriers through partner involvement, awareness creation, and service integration in all aspects of HIV/AIDS care. Variables associated with dual contraceptive utilization were having a child, having family support to use dual contraceptive methods, multiple sexual partners, and urban residence. The most common dual contraceptive used methods were condoms with injectables and condoms with pills.

## Supplementary Information

Below is the link to the electronic supplementary material.

Supplementary file1 (DOCX 43 kb)

## Data Availability

Data supporting this study can be obtained on request.
