# Correction to: Dual Contraceptive Method Utilization and Associated Factors Among HIV Positive Women Attending ART Clinic in Finote-Selam Hospital: Cross-Sectional Study

**DOI:** 10.1007/s10508-023-02613-7

**Published:** 2023-05-17

**Authors:** Anteneh Jemberie, Bewket Yeserah Aynalem, Liknaw Bewket Zeleke, Addisu Alehegn Alemu, Tenaw Yimer Tiruye

**Affiliations:** 1 Health Science College, Debre Markos University, 269 Debre Markos, Ethiopia; 2 School of Women’s and Children’s Health, University of New South Wales Sydney, Kensington, Australia; 3 Cancer Epidemiology and Population Health Research Group, Allied Health and Human Performance, University of South Australia, Adelaide, Australia

**Correction to: Archives of Sexual Behavior** 10.1007/s10508-023-02593-8

The name of coauthor Tenaw Yimer Tiruye has been corrected since the original publication of this article, and his place in the order of authorship has been corrected and an additional affiliation for him added.

The original article has been corrected.

