# Supplementary material for: Dual Contraceptive Method Utilization and Associated Factors Among HIV Positive Women Attending ART Clinic in Finote-Selam Hospital: Cross-Sectional Study
Source: Arch Sex Behav. 2023 Apr 7;52(6):2639–46. doi: 10.1007/s10508-023-02593-8 (PMC10501923; doi:10.1007/s10508-023-02593-8)
Supplement: Supplementary file 1 — Supplementary file1 (DOCX 43 kb) [file 10508_2023_2593_MOESM1_ESM.docx]

# Annex

## 1. English version questionnaires

Good morning/ Good afternoon, my name is__________. I am working as a data collector in this study that assesses Dual family planning utilization and associated factors among HIV-positive women attending care at Finoteselam Hospital You are kindly requested to participate in this study and provide the information required from you. Your participation in this study is completely voluntary bases and you have the right to refuse, to take part, or to interrupt at any time. But your participation will give us quite useful information to take dual family planning utilization among HIV positive having sexual intercourse in the last one month reproductive age women attending ART clinic. There are no incentives to participate in this study.

We would like to assure you, your name will not be mentioned anywhere. The information that you will give us will be kept confidential and only used for research purposes. The questionnaire will take 25-30 minutes. So are you willing to participate in this study?

Yes No

If the answer is yes, Thanks! Conduct

If the answer is no, skip to the next respondent.

Questionnaire code number_________________

Name of the data collector ______________Signature________ Date of interview_______

Name of the supervisor ____________Date of checking _________Signature__________

**Consent form**

I have read the information sheet and I understood the purpose and the expected benefit of the research of this study.

I hereby need to assure you with my signature that I have signed without any coercion & I have decided to participate voluntarily to take part in my contribution to the study which will help in decreasing both unintended pregnancy and STIs.

Signature______________ Date_______________

| **Part I. demographic factors** |
| --- |

| **S/no** | **Questions** | **Codes and answers** |
| --- | --- | --- |
| **101** | Age | _______years |
| **102** | Where is your residence? | 1. Urban 2.Rural |
| **103** | What is Your educational status? | 1.Can’t read & write 2.Only read and write(no grade)  3.Primary school(1-8) 4.Secondary school(9-12)  5. College and above |
| **104** | Which religion are you following? | 1 . Orthodox 2.Islam 3. Protestant 4. Catholic 5. Others |
| **105** | What is your ethnic group? | 1. Amhara 2.Oromo 3.Tigre 4.Gurage5.Others (specify)-- |
| **106** | Occupation | 1. Housewife 2. Merchant  3. Government employee 4. daily laborer 5. other(specify) |
| **107** | Marital status | 1. Married 2. Single 3. Divorced4. widowed |
| **108** | Average monthly income in BIRR | ------------------------------------- |

| **Part II: Reproductive & sexual related factor characteristics** |
| --- |

| **S/no** | Questions | Codes and answers | skip |
| --- | --- | --- | --- |
| **201** | What is Your age at 1^st^sex | ___________years |  |
| **202** | Do you have a child? | 1. Yes, 2. No | If no Q 204 |
| **203** | How many live children do you have? | 1. One child 2. two to four child   3. more than four children |  |
| **204** | Do you desire to have a child in the future | 1. Yes 2.No |  |
| **205** | Does your partner desire to have a child in the future? | 1. Yes, 2. No |  |
| **206** | Have you had sexual intercourse in the last 1 month? | 1. Yes, 2. No | If no Q 208 |
| **207** | Sex with whom? | 1. Regular partner(husband) 2. (multi-sexual partner) |  |
| **208** | Did you use condom/family planning methods? | 1. Yes, 2. No | If no Q 211 |
| **209** | How often did you use condom/family planning methods? | 1. Always 2. Some times |  |
| **210** | What was the reason for condom use? | 1. To prevent pregnancy  2. My partner is HIV negative  3. To reduce viral loads  4. fear of other STIs  5. Just health professionals' advice  6. other (specify) |  |
| **211** | Have you ever heard of any contraceptive methods that couples can use to avoid or delay pregnancy? | 1. Yes 2. No | If no Q213 |
| **212** | Which type of contraceptive method did you hear? | | |
| **212.1** | Pills | 1. Yes 2. No |  |
| **212.2** | Implant | 1. Yes 2. No |  |
| **212.3** | Injectable | 1. Yes 2. No |  |
| **212.4** | Condom | 1. Yes 2. No |  |
| **212.5** | IUDs | 1. Yes 2. No |  |

| **213** | | Do you know any contraceptive methods? | 1. Yes 2. No | | If no Q215 | |  |
| --- | --- | --- | --- | --- | --- | --- | --- |
| **214** | | Which types of contraceptive methods, did you know? | | | | |  |
| **214.1** | | Pills | 1. Yes 2. No | |  | |  |
| **214.2** | | Implant | 1. Yes 2. No | |  | |  |
| **214.3** | | Injectable | 1. Yes 2. No | |  | |  |
| **214.4** | | Condom | 1. Yes 2. No | |  | |  |
| **214.5** | | IUDs | 1. Yes 2. No | |  | |  |
| **215** | | Did you know sex without condom use risks STI transmission? | 1. Yes 2.No | |  | |  |
|  | | | | | | |  |
| **Part III፡ HIV related factor characteristics**   \| **301** \| Did your husband or partner get HIV tested? \| 1. yes 2.no \| If no  Q403 \| \| --- \| --- \| --- \| --- \| \| **302** \| What was his result? \| 1. positive 2. negative \|  \| \| **303** \| How long had since you have started ART? \| 1. <12 months 2. 12-24 months  3. >24 months \|  \| \| **304** \| How is your health status after starting ART? \| 1. Improved 2. same  3. worsened \|  \| \| **305** \| \| How much is your recent CD4 count? \| \| --- \| \| 1. CD4 count<250 cells/dl  2.CD4count250-350cells/dl  3.CD4count350-500cells/dl  4.>500cells/dl  5.. I didn’t remember \|  \|   **Part IV: dual contraceptive utilization** | | | | | | |  |
| **501** | Did you use a condom plus other contraceptives? | | | 1. Yes, 2. No | | If no Q 403 | |
| **502** | Which type of contraceptive do you use? | | | 1. Pill 2. Injectable 3. IUD   4. Permanent 5. Other specify | |  | |

##

Thank you

##

## 2. Amharic version questionnaires

እንደምንአደርሽ/ዋልሽስሜ---------------------------------ይባላል፡፡ የዚህ ጥናት መረጃ ሰብሳቢ ነኝ፡፡ ኮንዶምንና ሌላ የእርግዝና መከላከያ በአንድ ላይ የሚጠቀሙ እና ተዛማጅመንስኤዎቻቸዉን ለማወቅ በፍ/ሰላም ሆስፕታል የኤች .አይቪ /ኤድስ ቫይረስ በደመቸው ውስጥ ያላበቸው ፀረኤች አይቪ ህክምና በሚከታተሉ የግብረ ስጋ ግንኙነት ባለፈዉ አንድ ወር ወዲህ ባላቸዉ በመዉለድ እድሜ ክልል በሚገኙ እናቶች ላይ የሚከሄድ ጥናት ነው፡

**መግቢያ**

የዚህጥናት ማብራሪያና የስምምነት ቅጽ ዓላማ አሁን እርሰዎ እንዲሳተፉበት የምጠይቀዎተን የጥናት ምንነት ማብራራት ነው፡፡ በዚህጥናት ፕሮጀክት ለመሳተፍ ከመወሰንዎ በፊት ይህንን የማብራሪያ በቅጽ ሲነበብ በጥንቃቄበመገንዘብ ጥያቄዎች ካሎዎት ይጠይቁ፡፡ በተጨማሪም በጥናቱ መሳተፍ ከጀመሩ በኋላ በማንኛውም ጊዜ ጥያቄዎች ካሎዎት መጠየቅ ይችላሉ፡፡

ከ25-30 ደቂቃዎች በላይ እንደማይወስድ እገልፅሎታለሁ፡፡ ጥያቈዎቹን እጠይቆታለሁ ስሞዎት ከዚህ ቅጽ ላይ አይጠቀስም የሚሰጡኝ መረጃ በሚስጢር ይጠበቅሎታል ለአንዱ ወይም ለሁሉም ጥያቅዎች መልስ መስጠት ካልፈለጉ መብቶዎ የተጠበቀነው፡፡

ስለዚህለ ቃለ- መጠይቁ ፈቃደኛ ነዎት?

ፈቃደኛነኝ/ ፈቃደኛ አይደለሁም

መልሱአዎ ከሆነ ቃለ-መጠይቁን ይጀምሩ፡፡ አልፈልግም ከሆነ ቃለ-መጠይቁን አይጀምሩ፡፡

ፍቃደኛነቱን ያረጋገጠው መረጃ ሰብሳቢ

ስም----------------------------------------------------- ፊርማ-----------------------------

የመጠይቁመለያቁጥር------------------------------------

መጠይቁየተደረገበትቀን----------------------------------

የጥናቱተቆጣጣሪስም------------------------------------ ፊርማ------------------------------------

**የስምምነት መስጫ ቅፅ፡-**

እኔ ከዚህ በታች የፈረምኩት ስለ ጥናቱ ዓላማና ጥቅም ተረዴቸና አስፈላጊ መሆኑን አምኘበት በጥናቱ ላይ ለመሳተፍያለ ምንም ተፅዕኖ በራሴ ፈቃድ ተሳታፊ መሆኔን አረጋግጣለሁ፡፡

ፊርማ----------------------------ቀን-------------------------------

ተመራማሪዉን ማነጋገር ከፈለጉ በሚከተለው አድራሻ ማግኘት ይችላሉ፡-

**አንተነህ ጀምበሬ*.* ስልክ ቁጥር - 0918760397**

**ክፍል 1፡-የማህበራዊና ኢኮኖሚያዊ ሁኔታን የሚዲስሱ መጠይቆች**

| **ተ/ቁ** | **ጥያቄዎች** | **አማራጭ መልሶችና መለያኮድ** |
| --- | --- | --- |
| 101 | እድሜዎት ስንት ነው? | በዓመት--------------- |
| 102 | የሚኖሩበት ቦታ | 1.ከተማ 2.ገጠር |
| 103 | የትምህርት ደረጃዎት ምን ያህል ነው? | 1.ማንበብና መፃፍ የማይችሉ  2.ማንበብና መፃፍብቻ  3.የመጀመሪያ ደረጃ  4.የሁለተኛ ደረጃ 5.ኮሌጅ/ ዩኒቨርስቲ |
| 104 | የየትኛው ሃይማኖት ተከታይ ነዎት? | 1.ኦርቶድክስ 2.እስላም  3.ፕሮቴስታንት 4.ካቶሉክ 5.ሌላይገለፅ |
| 105 | ብሔረሰብዎ ምንድንነው? | 1.አማራ 2.ኦሮሞ  3.ትግሬ 4.ጉራጌ 5.ሌላ (ይገለፅ) |
| 106 | የሥራሁኔታ | 1.የቤትእመቤት2. ነጋዴ  3. የመንግስት ሰራተኛ  4. የቀንሰረተኛ 5. ሌለ ካለይገለፁ |
| 107 | የጋብቻሁኔታ | 1.ያገባች 2. ያላገባች  3. ባላየሞተባት 4.የፈታች |
| 108 | አማካኝ የወር ገቢዎ ምን ያህል ብር ነዉ | --------------------- ብር |

**ክፍሌ 2:-የውልደት እናየስነ-ተዋልዶ ታሪክ መጠይቅ**

| **ተ/ቁ** | **ጥያቄዎች** | **አማራጭ መልሶችና መለያ ኮድ** | **ይለፉ** |
| --- | --- | --- | --- |
| **201** | የመጀመረያ የግብረ ስጋ ግንኙነት ሲፈፅሙ ዕድሜዎ ስንት ነበር | _________ዓመት |  |
| **202** | ልጆችአለዎት | 1.አዎ 2.የለም |  |
| **203** | መልሶጥ.ቁ 202, አዎ ከሆነአሁን በህይወት ያሉት ስንት ናቸዉ | 1.አንድልጅ 2.ከሁለት እስከ አራት ልጆች  3. ከአራት ልጆችበላይ |  |
| **204** | ወደፊት ልጅእ ንድኖሮት ይፈልጋሉ | 1.አዎ 2.አልፈልግም |  |
| **205** | በላቤትሽ ወደፊት ልጅ እንድኖሮት ይፈልጋሉ | 1.አዎ 2.አይፈልግም |  |
| **206** | ባለፉት 1ወራት ውስጥ የግብረ-ሥጋግንኙነት ነበረዎት | 1.አዎ 2.አይደለም |  |
| **207** | መልሶቁጥር 206, አዎ ከሆነ የግብረ-ሥጋግንኙነት ከማንጋር ፈፀሙ? | 1.ከበለቤቴ ጋር  2.ከበለቤቴ ዉጭ ከብዙ ጎዳኛቼጋር |  |
| **208** | ኮንዶም/የእርግዝና መከላከያ ዘዴ ተጠቅመዋል? | 1. አዎ 2.አይደለም |  |

| **209** | መልሶ “ለጥ.ቁ # 208”, አዎ ከሆነ አጠቃቀምዎት እንዴትነው? | 1. ሁልጊዜ 2. አልፎአልፎ |  |
| --- | --- | --- | --- |

| **210** | ኮንዶም/የእርግዝና መከላከያ ዘዴ የተጠቀሙበት ምክንያት/ቶች ምንድን ነው/ናቸው (ከአንድበላይመልስይቻላል) | 1. እረግዝናንለመከላከል  2. ጓደኛዬ ከኤችአይቪ ቫይረስ ነፃ ስለሆነ  3. በሌላ አይነት የኤችአይቪ ቫይረስ  መያዝን ለመከላከል  4. ሌሎች የአበላዘር በሽታ ለመከላከል  5. የጤና ባለሙያ ምክር  6. ሌላ ካለይጥቀሱ---- |  |
| --- | --- | --- | --- |
| **211** | ስለ እርግዝና መከላከያ ዘዴ ከዚህ በፊት ሰምተዉ ያዉቃሉ? | 1. አዎ 2. አይደለም |  |
| **212** | መልሶ “ለጥ.ቁ # 211”, አዎ ከሆነ የትኛዉን እርግዝና መከላከያ ዘዴ ነው የሰሙት? | | |
| **212.1** | እንክብል | 1. አዎ 2. አይደለም |  |
| **212.2** | በክንድ የሚቀበር | 1. አዎ 2. አይደለም |  |
| **212.3** | በመርፌ የሚሰጥ የእርግዝና መከላከያ | 1. አዎ 2. አይደለም |  |
| **212.4** | ኮነንዶም | 1. አዎ 2. አይደለም |  |
| **212.5** | በማህፀን ዉስጥ የሚቀበር | 1. አዎ 2. አይደለም |  |
| **213** | የእርግዝና መከላከያ ዘዴወችን ያዉቃሉ? | 1.አዎ 2. አላዉቅም |  |
| **214** | መልሶ “ለጥ.ቁ # 213”, አዎ ከሆነ የትኛዉን የእርግዝና መከላከያ ዘዴ ያዉቃሉ? | | |
| **214.1** | እንክብል | 1. አዎ 2. አይደለም |  |
| **214.2** | በክንድ የሚቀበር | 1. አዎ 2. አይደለም |  |
| **214.3** | በመርፌ የሚሰጥ የእርግዝና መከላከያ | 1. አዎ 2. አይደለም |  |
| **214.4** | ኮነንዶም | 1. አዎ 2. አይደለም |  |

| **214.5** | በማህፀን ዉስጥ የሚቀበር | | 1. አዎ 2. አይደለም | |  |
| --- | --- | --- | --- | --- | --- |
| **215** | ኮንዶምን ሳይጠቀሙ የግብረስጋ ግንኙነት መፈጸም ለአባለዘር በሽታ እንደምያጋልጥ ያዉቃሉ? | | 1. አዎ 2. አይደለም | |  |
| **ክፍል 3፡ የኤች አይቪ ኤድስ ሁኔታ መጠይቅ**   \| **401** \| የትዳር አጋርዎ ከዚህ በፊት የደም ምርመራ አድርገዋል? \| 1. አዎ 2. አይደለም \|  \| \| --- \| --- \| --- \| --- \| \| **402** \| መልሶ “ለጥ.ቁ 401 አዎ ከሆነ የደም ምርመራዉ ጤቱ ምንነበር? \| 1.ፖዛቲቪ2.ኔጋቲቪ \|  \| \| **403** \| የፀረ ኤችአይቪ መድኃኒት ከጀመሩ ምን ያክል ጊዜ ሆነዎት? \| 1. <12 ወራት2. 12-24 ወራት  3. >24 ወራት  4. አላስተዉስም \|  \| \| **404** \| ፀረ ኤችአይቪ መድኃኒት ከጀመሩ ወድህ የጤንነትሽ ሁኔታ ምን ይመስላል? \| 1.ተሻሽሏል2.ያዉነዉ3.ብሶኛል \|  \| \| **405** \| በአሁኑ ጊዜ CD4 ስንት ነዉ? \| 1.ከ250 cells/dl በታች  2.ከ250-350cells/dl  3.ከ350-500cells/dl  4 ከ500cells/dl በላይ  5.አላስተዉስም \|  \|   **ክፍል 5. ሁለት የቤተሰብ ምጣኔ አጠቃቀም መጠይቅ** | | | | | |
| **501** | ከኮንዶም በተጨማሪ ሌላ የቤተሰብ ምጣኔ አገልግሎት ይጠቀማሉ? | 1. አዎ 2.አይደለም | |  | |
| **502** | መልሶ “ለጥ.ቁ # 501”, አዎ ከሆነ የትኛዉን አይነት የቤተሰብ ምጣኔ አገልግሎት ይጠቀመሉ? (ከአንድ በላይ መልስ ይቻላል) | 1. እንክብል  2. በመርፉ የሚሰጥ የእርግዝና መከላከያ  3. በማህጸን ዉስጥ የምቀመጥ  4.ቋሚ የእርግዝና መከላከያ  5.ሌሎች ካሉ ይጥቀሱ | |  | |

//አመሰግናለሁ//
